# A Long-Term and Slow-Releasing Hydrogen Sulfide Donor Protects against Myocardial Ischemia/Reperfusion Injury

**DOI:** 10.1038/s41598-017-03941-0

**Published:** 2017-06-14

**Authors:** Xiaotian Sun, Wenshuo Wang, Jing Dai, Sheng Jin, Jiechun Huang, Changfa Guo, Chunsheng Wang, Liewen Pang, Yiqing Wang

**Affiliations:** 1 Department of Cardiothoracic Surgery, Huashan Hospital of Fudan University, Shanghai, 200040 China; 2 Department of Cardiac Surgery, Zhongshan Hospital of Fudan University and Shanghai Institute of Cardiovascular Diseases, Shanghai, 200032 China; 3 Department of Cadres Health Care, Third Hospital of Shijiazhuang, Shijiazhuang, 050011 China; 4 Department of Physiology, Hebei Medical University, Shijiazhuang, 050017 China

## Abstract

Hydrogen sulfide (H_2_S) has been recognized as an important gasotransmitter exerting various physiological effects, especially in the cardiovascular system. Herein we investigated the cardioprotective effects of a novel long-term and slow-releasing H_2_S donor, DATS-MSN, using *in vivo* myocardial ischemia/reperfusion (I/R) models and *in vitro* hypoxia/reoxygenation cardiomyocyte models. Unlike the instant-releasing pattern of sodium hydrosulphide (NaHS), the release of H_2_S from DATS-MSN was quite slow and continuous both in the cell culture medium and in rat plasma (elevated H_2_S concentrations during 24 h and 72 h reperfusion). Correspondingly, DATS-MSN demonstrated superior cardioprotective effects over NaHS in I/R models, which were associated with greater survival rates, reduced CK-MB and troponin I levels, decreased cardiomyocyte apoptosis index, increased antioxidant enzyme activities, inhibited myocardial inflammation, greater reduction in the infarct area and preserved cardiac ejection fraction. Some of these effects of DATS-MSN were also found to be superior to classic slow-releasing H_2_S donor, GYY4137. In *in vitro* experiments, cardiomyocytes injury was also found to be relived with the use of DATS-MSN compared to NaHS after the hypoxia/reoxygenation processes. The present work provides a novel long-term and slow-releasing H_2_S donor and an insight into how the release patterns of H_2_S donors affect its physiological functionality.

## Introduction

Hydrogen sulfide (H_2_S) is a novel gasotransmitter that can exert various physiological and pathophysiological effects, particularly in the cardiovascular system. Increasing evidences suggest that the production of endogenous H_2_S or the administration of exogenous H_2_S can successfully attenuate myocardial infarction (MI) following ischemia and reperfusion (I/R) injury. Sodium hydrosulphide (NaHS), the most commonly used H_2_S donor, can reduce the myocardial infarct size and preserve left ventricular (LV) function following I/R injury in both preconditioning or postconditioning experiments^1, 2^. However, the instant release of H_2_S from NaHS cannot mimic the slow and continuous process of H_2_S generation *in vivo* ^3^. The rapid production and loss of H_2_S may also lead to imprecise experimental results and detrimental effects in the body (e.g., hypotension and neurotoxicity)^4^. Furthermore, the unstable features of NaHS in an aqueous solution make it difficult to be reformulated by drug carriers, such as nanoparticles. Therefore, many other long-term release compounds have emerged as H_2_S donors, including morpholin-4-ium 4 methoxyphenyl (morpholino) phosphinodithioate (GYY4137)^3^, cysteine analogs^5^, dithiolethione and its chimeras^6^, and cysteine-activated H_2_S donors^7^. However, the release processes of these H_2_S donors are hardly regulated, and the H_2_S concentrations are relatively low both *in vitro* and *in vivo*. Recently, a natural garlic-derived polysulfide compound–diallyl trisulfide (DATS) has drawn increased attention as a potential H_2_S donor, regarding its ability to generate H_2_S in the presence of reduced glutathione (GSH) both in red blood cells and phosphate buffers (PBS)^8^. Furthermore, the beneficial effects of DATS for cardiovascular diseases are also shown to be derived from H_2_S^9^. However, the poor solubility of DATS in aqueous media limits its use as an H_2_S donor, and its H_2_S release still remains relatively rapid after the addition of GSH^10^.

Recently, by exploiting mesoporous silica nanoparticles (MSN) as the carrier of DATS, a novel GSH-activated, water-dispersible, slow and controllable releasing H_2_S system (DATS-MSN) was synthesized in our laboratory (*Supporting Information*, Fig. S1). Unlike NaHS, DATS is quite stable in an aqueous solution with minimal spontaneous H_2_S release, which makes it an excellent drug to be loaded and reformulated. Due to the unique mesoporous structures of MSN and its loading ability, the new H_2_S donor DATS-MSN can be activated by GSH with slow and controllable H_2_S release rates^10^. Therefore, DATS-MSN presents a much slower process of H_2_S generation than free DATS, both *in vitro* and *in vivo* with excellent biocompatibility, which makes it an ideal slow-releasing H_2_S donor^10^. The present study aimed to investigate the cardioprotective effects of DATS-MSN treatment in a rat I/R model compared with the classic H_2_S donors NaHS and DATS, as well as the slow-releasing H_2_S donor GYY4137, and to explore how the different release patterns of H_2_S affect its physiological functions.

## Methods

### Materials

Newborn (6 g, 24 h) and adult male Sprague-Dawley rats (250–280 g, 8 w) were used in this study. The adult rats were housed under a 12-h/12-h light/dark cycle with food and water provided *ad libitum* during the experimental protocol. All animal experiments were approved by Institutional Review Board and Institutional Animal Care and Use Committee Protocols of Fudan University, and confirmed with the *Guide for the Care and Use of Laboratory Animals* published by the US National Institutes of Health (NIH publication no. 85–23, revised 1996). DATS-MSN was synthesized and characterized by the method described previously^10^ (*Supporting Information*). NaHS, GSH, DATS, GYY4137 and other chemical reagents were obtained from Sigma-Aldrich.

### *In vitro* Cell Viability and Cytotoxicity Assay after Hypoxia/reoxygenation Procedure

The isolation and culture of primary neonatal cardiomyocytes were performed using the method described previously^10^ (*Supporting Information)*. Cardiomyocytes were divided into seven groups (n = 3): Control group: same volume of PBS (normoxia); Vehicle group: same volume of PBS; GSH group: GSH (2 mM); MSN group: MSN (5 μg/mL); NaHS group: NaHS (80 μM); DATS group: DATS (5 μg/mL) + GSH (2 mM); DATS-MSN group: DATS-MSN (10 μg/mL) + GSH (2 mM). The concentrations of NaHS and DATS were selected based on their most effective concentrations described in the *Supporting Information* (Fig. S2). The concentration of DATS-MSN was determined to compare the amounts of total sulfur atoms with that in the NaHS and DATS groups (S atoms: 80 μM of NaHS *vs*. 84.3 μmol/kg of DATS *vs*. 74.2 μmol/kg of DATS-MSN).

The culture medium of each group (except for the Control group) was removed and replaced with DMEM/F-12 without glucose and serum, and the cells were exposed to hypoxic conditions (94% N_2_, 5% CO_2_, 1% O_2_) for 4 h in a CO_2_ incubator (Forma SERIES II WATER JACKET, Thermo Scientific, MA, USA), followed by reoxygenation (95% O_2_, 5% CO_2_) with fetal bovine serum for 6 h. Drugs were administrated at the time of reoxygenation, and cell viability, lactate dehydrogenase (LDH) activity and creatine kinase (CK) activity were evaluated within 6 h following reoxygenation. The H_2_S concentrations in the culture medium were also measured using a high-performance liquid chromatography (HPLC) method as described previously^10^ (*Supporting Information*).

After the hypoxia/reoxygenation procedure, the culture medium was removed; the cells were washed with PBS, and then resuspended in cell counting kit-8 (CCK-8) solution (Dojindo Laboratories, Kumamoto, JA) as the medium. The absorbance of the individual wells was measured at 450 nm via a microplate reader (Molecular Devices, FlexStation 3, CA, USA). The results were expressed as the mean percentage of cell viability relative to the Control group. The LDH and CK activities for each group were also measured using an assay kit (JianCheng, Nanjing, China) in accordance with the manufacturer’s instructions.

### *In Vivo* Myocardial I/R Model

Male Sprague-Dawley rats undergoing the I/R protocol were randomly assigned into five groups (n = 6, counting alive animals): Vehicle group: 500 μL of PBS; MSN group: 2 mg/kg of MSN; NaHS group: 30 μmol/kg of NaHS; DATS group: 2 mg/kg of DATS; and DATS-MSN group: 4 mg/kg of DATS-MSN. There were six extra rats undergoing an open-close chest procedure that comprised the Sham group (500 μL of PBS). The dosages of NaHS and DATS were chosen based on their most effective dosages for the reduction of myocardial injury during the I/R process, which is described in the *Supporting Information* (Fig. S3). The dose of DATS-MSN was determined by comparing the amounts of total sulfur atoms with NaHS and DATS administrated (S: 30 μmol/kg of NaHS *vs*. 33.7 μmol/kg of DATS *vs*. 29.7 μmol/kg of DATS-MSN).

### Ischemic/reperfusion protocol

The I/R model was induced by ligating the left anterior descending coronary artery (LAD) as previously described^1^. Briefly, the rats were anesthetized with medetomidine hydrochloride (Domitor, 250 μg/kg, *ip*.) and ketamine hydrochloride (Ketamine, 50 mg/kg, *ip*.), followed by an endotracheal intubation. The carotid arteries were cannulated for blood withdrawal and monitoring of the blood pressure and heart rates. The LAD was temporarily ligated using a 6–0 silk suture 3 mm from its origin between the artery conus and the left atrium. Successful ligation of the coronary artery was verified by the color change in the ischemic area (anterior ventricular wall and the apex) of the heart. The rats were subjected to 30 min of ischemia followed by 24 h reperfusion. All drugs were administrated via a tail vein injection at the time of reperfusion. The mean arterial pressure (MAP) and heart rates were recorded at baseline, 30 min after ischemia, as well as 30 min and 6 h after reperfusion; the arrhythmia scores were also calculated at the same time point as described in the previous study^11^ (*Supporting Information*). The survival rates were evaluated based on the animals surviving throughout the experimental protocol until 24 h after reperfusion. At 24 h after reperfusion, the hearts were excised, washed, and stored for subsequent experimental analysis.

### Determination of Myocardial Injury and the H_2_S Concentration

The levels of creatine kinase-MB (CK-MB) and cardiac troponin I were measured to indicate and evaluate the extent of myocardial injury. Blood samples from the rats were obtained at 24 h after reperfusion just before death, and analyzed using Analytics (Gaithersburg, MD, USA). The H_2_S concentrations in plasma were evaluated within the first 12 h and at 24 h after reperfusion before death, and the H_2_S concentrations in the myocardium were also evaluated immediately after heart excision by HPLC as previous described^10^ (*Supporting Information*).

### Transferase-Mediated dUTP Nick-End Labeling (TUNEL) Assay

A piece of ischemic heart tissue was excised, fixed in 4% paraformaldehyde, paraffin-embedded, sectioned, and stained with hematoxylin-eosin (H&E), followed by a TUNEL assay: the cell nuclei were stained with 4′,6′-diamidino-2-phenylindole hydrochloride (DAPI) color development kits (Roche, Basil, CH) in accordance with the manufacturers’ instructions. The cell nuclei that stained green were defined as TUNEL-positive nuclei and were monitored using a fluorescence microscope (Olympus IX-71, Japan). The proportion of TUNEL positive nuclei per 500 nuclei was quantified at 200x magnification.

### Western Blot Assay

A piece of ischemic heart tissue was homogenized by a rotor-stator homogenizer in ice-cold RIPA buffer (Pierce, Pittsburgh, PA, USA), and incubated at 4 °C overnight. After boiling with loading buffer (Fermentas, Glen Burnie, MD, USA), denatured proteins were separated in SDS PAGE gel, and transferred onto PVDF membrane. The membrane was blocked with 5% nonfat milk, followed by incubation with primary antibody of Bcl-2 and Bax (Abcam, Cambridge, MA, USA) at 4 °C overnight. HRP-conjugated secondary antibody (Kangchen Bio-tech, Beijing, China) was used to incubate the membrane for another 2 h. SuperSignal West Pico Chemiluminescent Substrate (Pierce, Pittsburgh, PA, USA) was poured on the membrane to develop the band captured by FluorChem Image System (Alpha Innotech, Santa Clara, CA, USA).

### Determination of Caspases-3 Activity

The activity of caspase 3 in the border zone of infarcted area was determined using the colorimetric assay via a microplate reader at 400 nm. The assay kits were purchased from Biovision (Milpitas, CA).

### Antioxidant Enzyme Activities

A total of 50 mg ischemic heart tissue was homogenized in a 50 mM ice-cold potassium phosphate buffer (pH of 6.8). Superoxide dismutase (SOD) activity was measured as described by Dieterich^12^, which was determined by monitoring the inhibition of pyrogallol autooxidation at 420 nm. The catalase (CAT) activity was determined by the modified method of Alvarez^13^. The GSH levels were measured by using a commercially available kit according to the manufacturer’s instructions (Beytime Institute of Biotechnology, Nantong, China). The malonydialdehyde (MDA) levels were measured using the thiobarbituric acid (TBA) assay, with 1, 1, 3, 3-tetramethoxypropane as an external standard for the construction of standard curves. The activity of SOD and CAT, and the levels of MDA and GSH were all standardized by protein content, determined using a bicinchoninic acid (BCA) protein assay kit (Beytime Institute of Biotechnology, Nantong, China).

### Quantitative Assessment of Neutrophil Accumulation

Ischemic heart tissues were assessed for the myeloperoxidase (MPO) activity as a marker of neutrophil accumulation. Tissues were homogenized in a solution containing 0.5% hexadecyltrimethylammonium bromide dissolved in 10 mM K_3_PO_4_ buffer (pH of 7) and centrifuged for 30 min (20, 000 g, 4 °C). An aliquot of the supernatant was allowed to react with a solution of tetramethylbenzidine (1.6 mM) and 0.1 mM H_2_O_2_. The change in absorbance was measured by spectrophotometry at 650 nm. The MPO activity was defined as the quantity of enzyme degrading 1 mmol of hydrogen peroxide per minute at 37 °C and expressed in milliunits per milligram protein.

### Measurement of cytokines

Blood was collected at 24 h after reperfusion before the animals were sacrificed. The samples were centrifuged, and then the plasma was collected according to the manufacturer protocol. The expressions of TNF-a and IL-1β cytokines were measured with an ELISA Kit (R&D Systems).

### Determination of the Infarct Size and Cardiac Function at 72 h Reperfusion

Repeated I/R protocol (n = 6) and then evaluated the infarct size and cardiac function at 72 h after reperfusion. The rats were anesthetized again after 72 h of reperfusion, and the LAD was ligated again in the same location. Evans blue (5.0%, 1.0 mL) was injected into the carotid artery to delineate between the ischemic and nonischemic zones. Then, the heart was rapidly excised and cross-sectioned into 1-mm-thick sections, and incubated in a 1% triphenyltetrazolium chloride (TTC) solution for 30 min. The stained slices were photographed with a digital camera, and infarct zones were quantified as a percentage of the total myocardium using Image J software (NIH, Boston, USA). The myocardial area at risk (AAR) and infarct per left ventricle (INF) were determined by a blinded observer.

Cardiac function was evaluated before the animals were sacrificed using an echocardiography method. The ejection fraction (EF), fractional shortening (FS), left ventricular internal dimension in diastole (LVIDd), and the left ventricular internal dimension in systole (LVIDs) were obtained and compared with the baseline values before the infarction (*Supporting Information*). The H_2_S concentrations in plasma and myocardium were also evaluated after 72 h reperfusion just before the sacrifice as previously described^10^.

### Comparison of DATS-MSN to Classic Slow-releasing H_2_S Donor in Myocardial Protection

To compare myocardial protective effects of the novel slow-releasing H_2_S donor to traditional slow-releasing donors, the most popular and classic H_2_S slow-releasing donor GYY4137 was utilized. The *in vivo* experiments above were repeated, and the protective effects such as myocardial injury protection, anti-apoptosis, antioxidant, anti-inflammation (at 24 h after reperfusion), and reducing infarct size and preserving cardiac function (at 72 h after reperfusion) were evaluated in two H_2_S slow-releasing donors. The dose of GYY4137 (100 mg/kg) was chosen based on its most effective dose for the reduction of myocardial injury during the I/R process, which is described in the *Supporting Information* (Fig. S3). The *in vivo* releasing feature of H_2_S of GYY4137 was also determined using HPLC as previous described^10^.

### Statistical Analysis

The data are expressed as mean ± SEM. One-way analysis of variance (ANOVA) was used to examine statistical comparisons between groups. The significant difference between two groups was analyzed by Student’s t test. The Chi-square test (or Fisher’s exact test when appropriate) was used to compare discrete variables between different groups. A value of P < 0.05 was considered to be significant. All authors had full access to, and take full responsibility for the integrity of the data.

### Data Availability

The datasets generated during and/or analysed during the current study are available from the corresponding author on reasonable request.

## Results

### DATS-MSN Protected Rat Cardiomyocytes from Hypoxia/Reoxygenation Induced Damage

**Figure 1:**
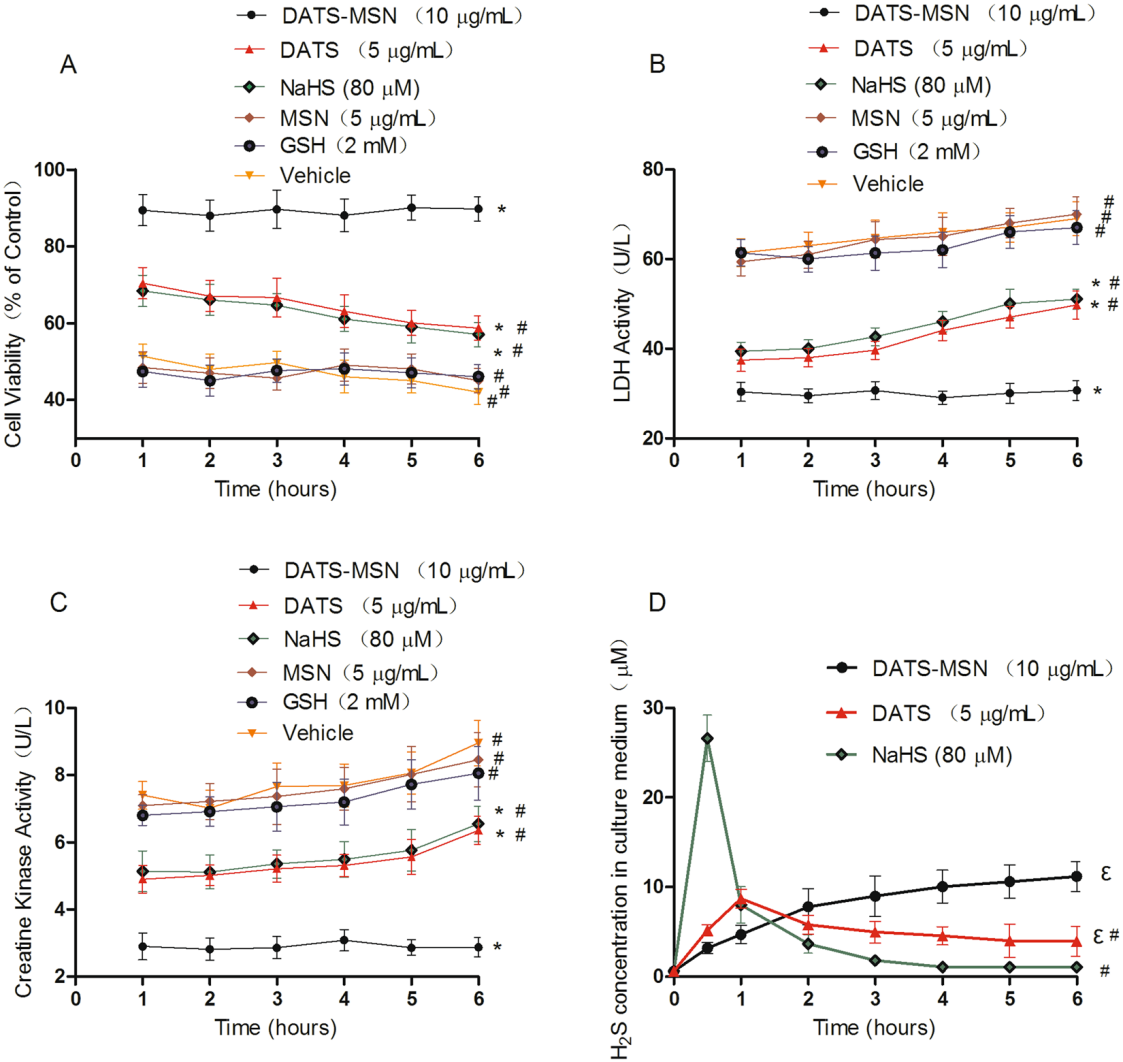
Protective effects of different H_2_S donors from hypoxia/reoxygenation induced damage in rat cardiomyocytes: Cell viability (**A**), lactate dehydrogenase (LDH) activity (**B**), creatine kinase activity (**C**) and H_2_S concentration in culture media (**D**) were evaluated within 6 h reoxygenation procedure. *P < 0.05 compared with the Vehicle at 6 h; ^#^P < 0.05 compared with the DATS-MSN group at 6 h; ε: P < 0.05 compared with the NaHS group at 6 h (mean ± SEM, n = 6).

As shown in Fig. 1A–C, all three H_2_S donors effectively protected cardiomyocytes from hypoxia/reoxygenation injury, with greater cell viability and decreased LDH and CK activity observed at 6 h after reoxygenation. However, the DATS-MSN group exhibited an obvious advantage with regards to cellular protection compared with the NaHS and DATS groups. This advantage became even more significant along with the reoxygenation time expanding. Moreover, Fig. 1D showed that the *in vitro* H_2_S release from NaHS was quite rapid, peaked at 30 min, and rapidly decreased to nearly baseline levels after 3 h without reaching a plateau. In contrast, the release of H_2_S from DATS-MSN was much slower and continuous. In addition, the curve continued to increase after the 6 h experiment. The DATS release curve was between NaHS and DATS-MSN, peaking at nearly 1 h. There was no obvious difference between the Vehicle, GSH, and MSN groups, suggesting that the cytoprotective effects were not be mediated by the administration GSH or MSN.

### DATS-MSN Improved the Hemodynamics and Survival during the I/R Process

**Figure 2:**
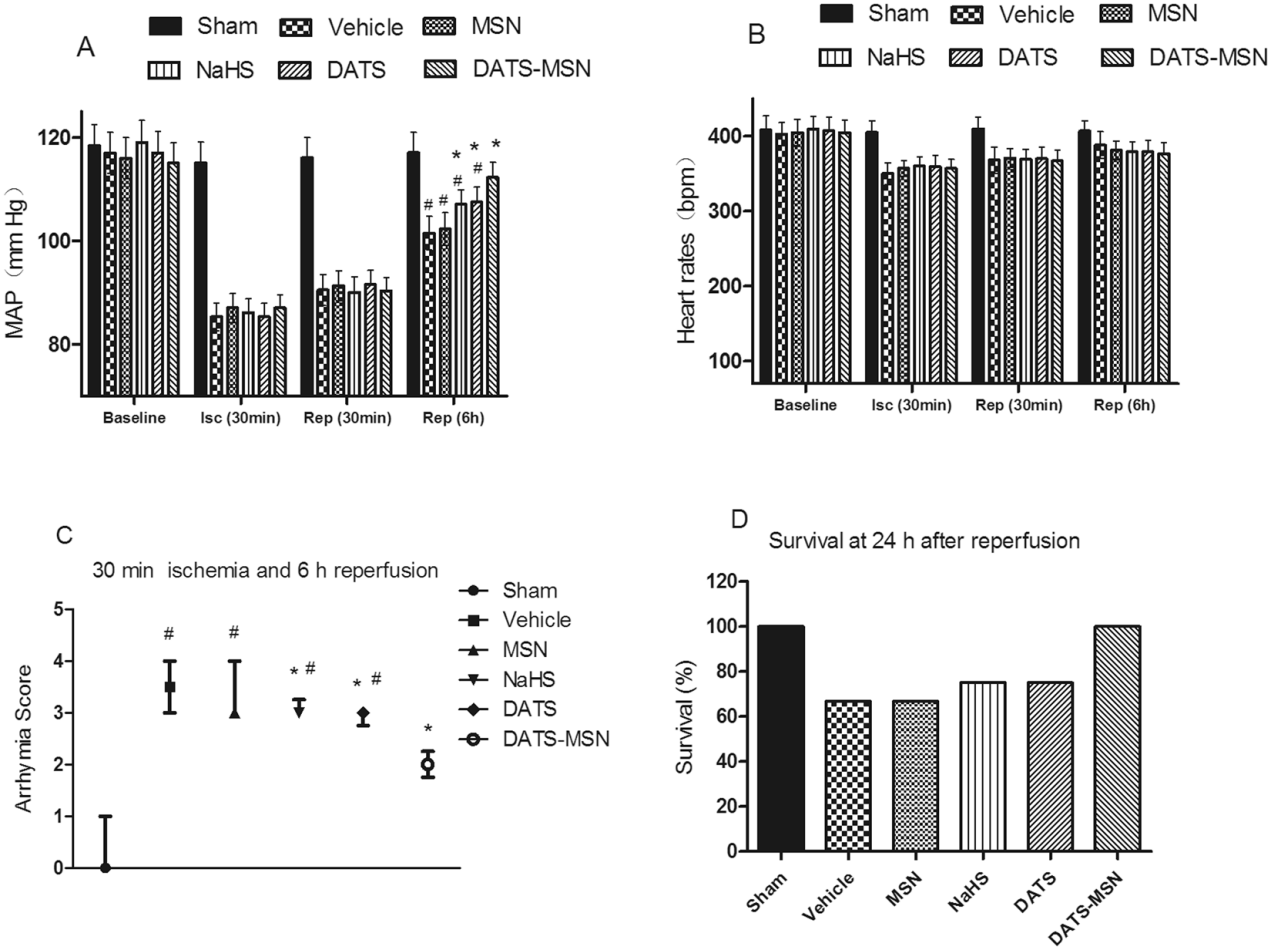
Hemodynamics alterations and survival during the ischemia and reperfusion process: mean arterial pressure (MAP) (**A**), heart rates (**B**) and in arrhythmia score (**C**) in 30 min ischemia and 6 h reperfusion process; and survival (**D**) at 24 h after reperfusion. *P < 0.05 compared with the Vehicle; ^#^P < 0.05 compared with the DATS-MSN group (mean ± SEM, n = 6).

As shown in Fig. 2A, the H_2_S donor groups exhibited elevated MAP levels compared with the Vehicle group at 6 h after reperfusion, which was most obvious in the DATS-MSN group; however, there was no difference in the alteration in the heart rates between groups (Fig. 2B). The DATS-MSN was also associated with substantially lower arrhythmia scores compared with the NaHS and DATS groups during the 30 min ischemia and 6 h reperfusion period (Fig. 2C). At 24 h after reperfusion, the survival was 100% in the Sham and DATS-MSN groups, compared to 66.7% (6 of 9 rats) in the Vehicle and MSN groups, and to 80.0% (6 of 8 rats) in NaHS and DATS groups (Fig. 2D). The main cause of death was due to lethal ventricular arrhythmias occurring after reperfusion.

### DATS-MSN Reduced Myocardial Injury after the I/R Process

**Figure 3:**
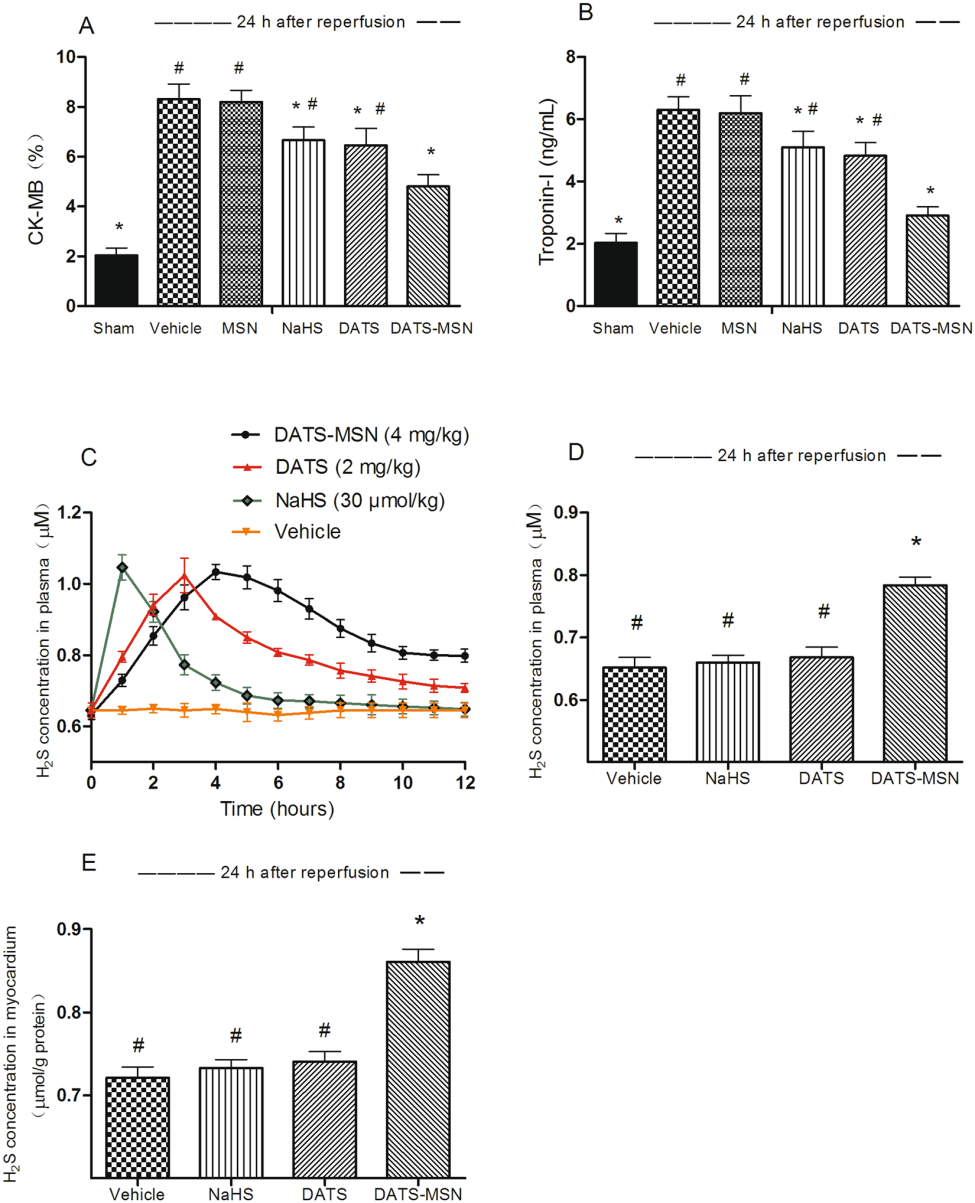
Myocardial injury evaluation and *in vivo* measurement of H_2_S levels during 24 h reperfusion process. Changes in serum creatine kinase MB (CK-MB) (**A**) and Troponin-I (**B**) levels at 24 h after reperfusion; H_2_S concentration in plasma after tail vein injection of NaHS (30 μmol/kg), DATS-MSN (4 mg/kg), DATS (2 mg/kg) and same volume of PBS during 12 h reperfusion (**C**); H_2_S concentrations were also measured in plasma (**D**) and in myocardium (**E**) at 24 h after reperfusion; drugs were administrated at beginning of reperfusion. *P < 0.05 compared with the Vehicle group; ^#^P < 0.05 compared with DATS-MSN group (mean ± SEM, n = 6).

As shown in Fig. 3A and B, the CK-MB and troponin I levels were reduced by three H_2_S donors at 24 h after reperfusion, among which the DATS-MSN group exhibited the greatest decrease in these biomarkers of myocardial injury. Following the intravenous injection of three H_2_S donors with a similar amount of total sulfur atoms (S: 30 μmol/kg of NaHS *vs*. 29.7 μmol/kg of DATS-MSN *vs*. 33.7 μmol/kg of DATS), the H_2_S concentration in plasma of the DATS-MSN group increased from the first time point (1 h), peaked at 4 h after administration, and remained elevated over the course of the 12 h experiment. In contrast, NaHS rapidly increased the plasma H_2_S concentration after administration (peaking at 1 h), which, however, quickly decreased to near baseline levels after 6 h (Fig. 3C). DATS elevated the plasma H_2_S concentration more rapidly than DATS-MSN, but slowly than NaHS, peaking at nearly 3 h after administration. Furthermore, the H_2_S levels in plasma and heart tissue remained elevated in the DATS-MSN group following the 24 h reperfusion process, which did not occur in NaHS, DATS, and Vehicle groups at this time point (Fig. 3D and E).

### DATS-MSN Protected against Myocardial Apoptosis

**Figure 4:**
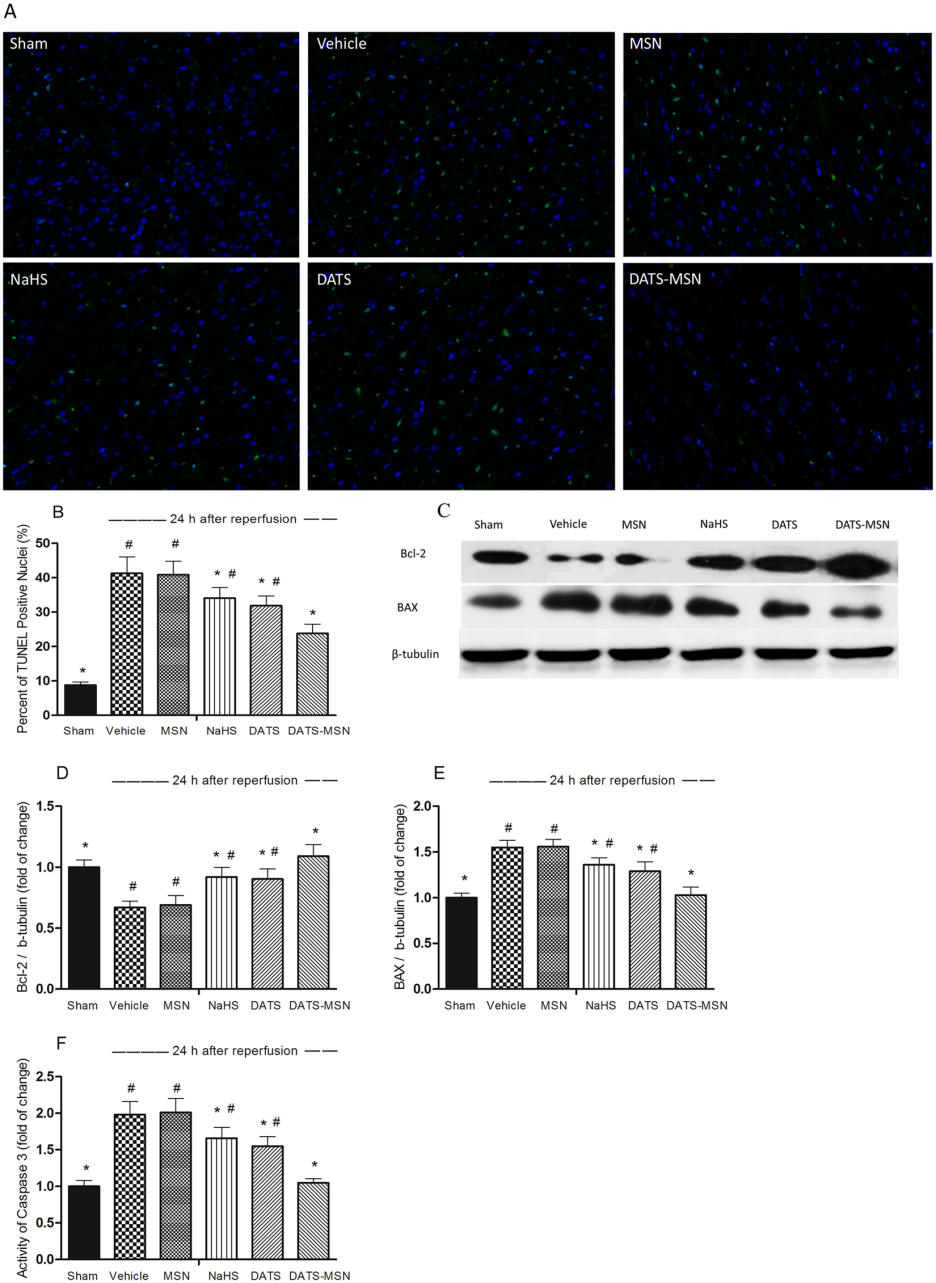
The changes of cardiomyocytes apoptosis and related factors with apoptosis after 24 h reperfusion. (**A**) TUNEL staining detected cardiomyocytes apoptosis. Nuclei with green staining indicate TUNEL positive cells (200 × ); (**B**) the percentage of TUNEL positive cells to total cardiomyocytes. The level of Bcl-2 and Bax in ischemia area were test using western blot assay (**C**); the intensity of each band was quantified by densitometry, and data were normalized to the β-tubulin signal (**D and E**); the level of Caspase-3 activity (**F**) was test using ELISA assay. *P < 0.05 compared with the Vehicle; ^#^P < 0.05 compared with the DATS-MSN group (mean ± SEM, n = 6).

Figure 4A presents the representative TUNEL results following 24 h of reperfusion. Although the apoptosis index was significantly decreased in all the H_2_S donor groups, the DATS-MSN was associated with the most potent anti-apoptosis ability among the three H_2_S donors (Fig. 4B).

As shown in Fig. 4C–E, the I/R injury induced the expression of Bax and reduced the expression of Bcl-2 in the Vehicle group. The NaHS, DATS, and DATS-MSN groups all exhibited increased levels of Bcl-2, but decreased levels of Bax, respectively, providing protection against myocardial apoptosis. These effects were also most remarkable in the DATS-MSN group. Accordingly, the Caspase-3 activity was associated with similar results, exhibiting the greatest activity decrease in the DATS-MSN group (Fig. 4F).

### DATS-MSN Resisted Oxidative Stress following the I/R Protocol

**Figure 5:**
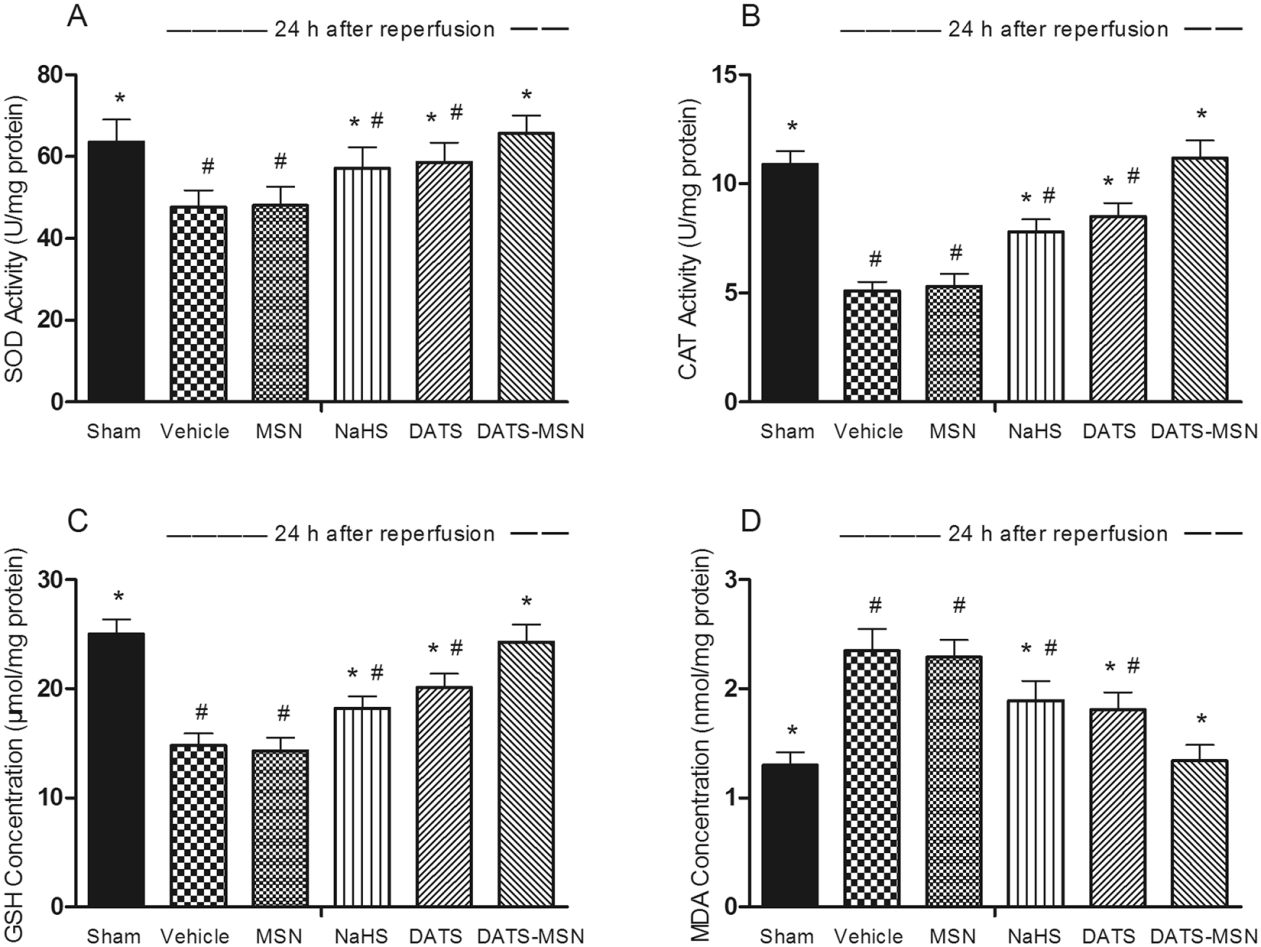
Levels of antioxidant defensive enzymes and malonydialdehyde (MDA) levels in heart tissues after ischemia/reperfusion injury. (**A**) Superoxide dismutase (SOD) activities, (**B**) catalase (CAT) activities, (**C**) levels of glutathione (GSH) and (**D**) levels of MDA contents in ischemia heart tissue after 30 min ischemia and 24 h reperfusion.*P < 0.05 compared with the Vehicle; ^#^P < 0.05 compared with the DATS-MSN group (mean ± SEM, n = 6).

As shown in Fig. 5, the NaHS, DATS and DATS-MSN groups all displayed an effective preservation of SOD activity, CAT activity, and GSH levels compared with the Vehicle group, and DATS-MSN exerted more significant antioxidant effects than the other two H_2_S donors (Fig. 5A–C). In addition, the tissue levels of MDA, an important biomarker of oxidative stress injury, were also found to be much lower in the DATS-MSN group compared to the NaHS and DATS groups (Fig. 5D).

### DATS-MSN Reduced Myocardial Inflammation following the I/R Protocol

**Figure 6:**
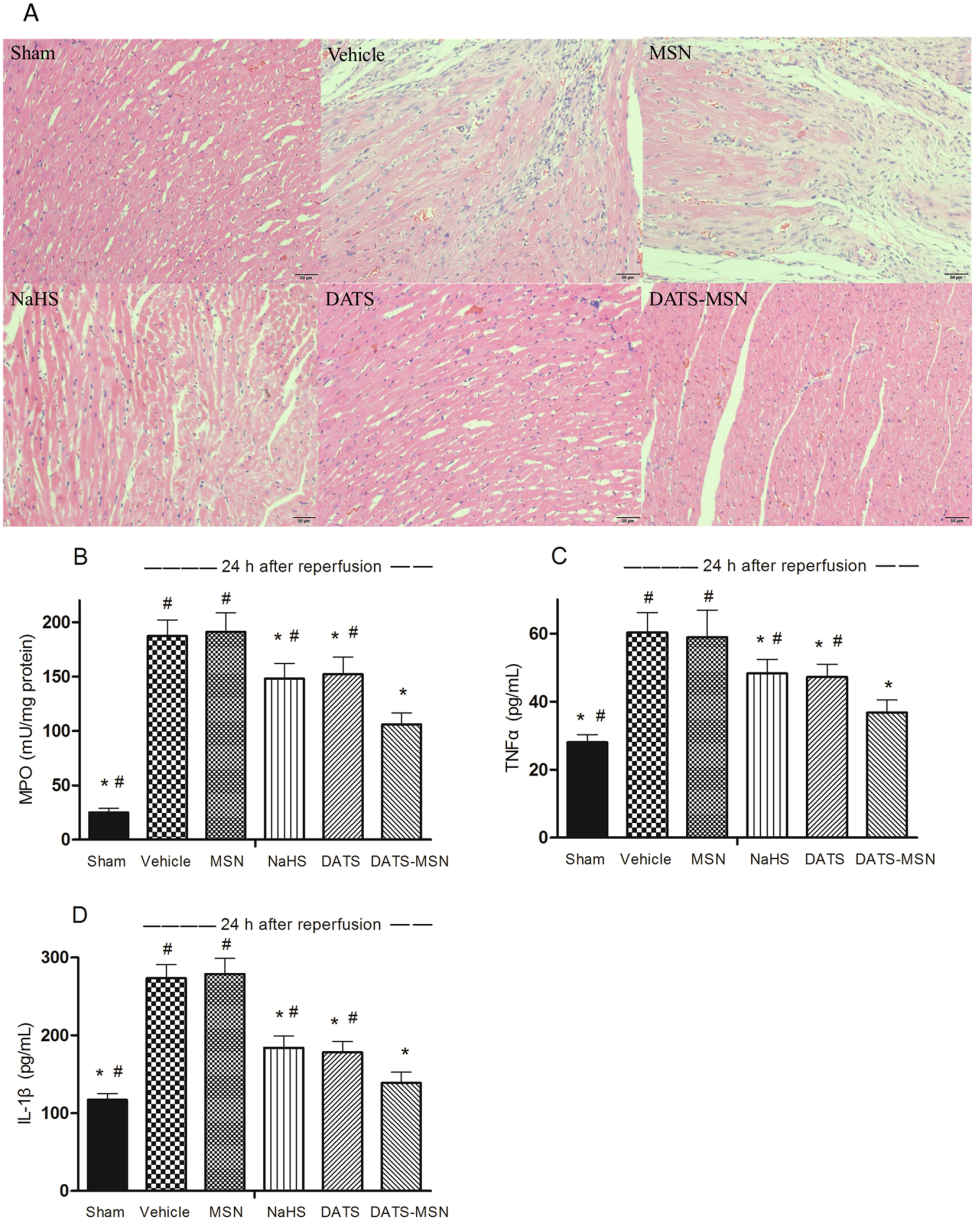
Myocardial inflammation levels after ischemia/reperfusion injury. Representative H&E-stained histological images (**A**), myeloperoxidase (MPO) activity (**B**), TNF-α (**C**) and IL-1β (**D**) levels after 30 min ischemia and 24 h reperfusion. *P < 0.05 compared with the Vehicle; ^#^P < 0.05 compared with the DATS-MSN group (mean ± SEM, n = 6).

Heart sections stained with H&E were presented at 24 h after reperfusion (Fig. 6A). The rats receiving H_2_S donors displayed a reduced degree of myocardial neutrophilic infiltrate and necrosis in comparison with the Vehicle group, with a reduction in MPO activities (Fig. 6B). Furthermore, the histological analysis and MPO activity assessment both revealed that DATS-MSN exerted the most potent anti-inflammatory effects among the three H_2_S donors. The serum levels of TNF-α and IL-1β measured at 24 h after reperfusion also revealed that the most significant decrease was observed in the DATS-MSN group compared with the other two H_2_S donor groups and the Vehicle group.

### DATS-MSN Reduced Infarct Size and Preserved Cardiac Function after 72 h of Reperfusion

**Figure 7:**
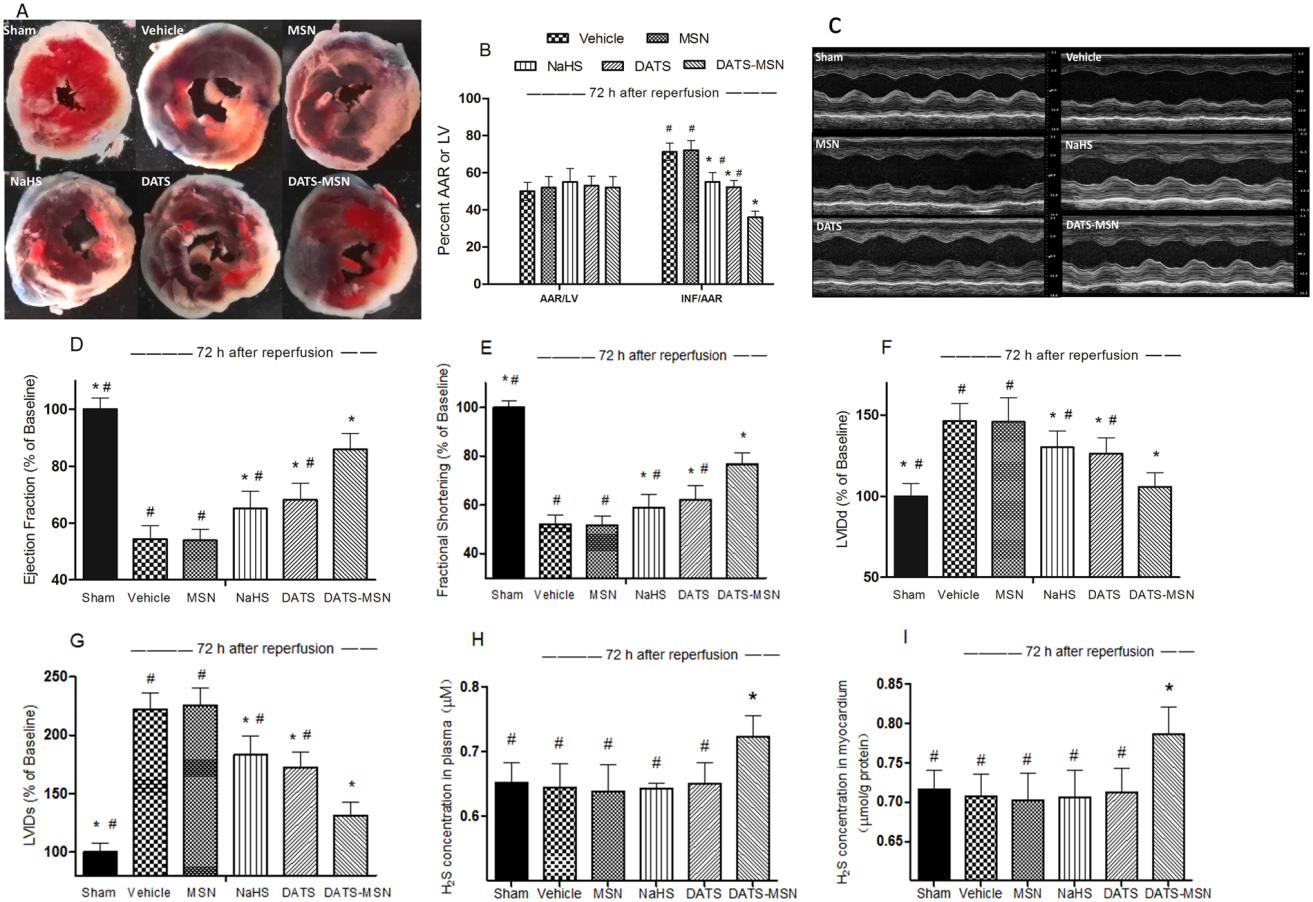
Myocardial infarction size and cardiac function at 72 h after reperfusion. (**A**) Representative mid-myocardial cross sections of TTC-stained hearts at 72 h after reperfusion. Dark blue area (Evan’s blue-stained): nonischemic zone; remaining area: AAR; white area: infracted tissue; red area (TTC-positive): viable myocardium; (**B**) percentage of area at risk (AAR) to total LV, and infarction area (INF) to AAR at 72 h after reperfusion. Representative M-mode images from individual groups (**C**), ejection fraction (**D**), fractional shortening (**E**), left ventricular internal dimension in diastole (LVIDd) (**F**) and left ventricular internal dimension in systole (LVIDs) (**G**) were measured by M-mode echocardiography. H_2_S concentrations in plasma (**H**) and in myocardium (**I**) were measured at 72 h after reperfusion. *P < 0.05 compared with the Vehicle group; ^#^P < 0.05 compared with DATS-MSN group (mean ± SEM, n = 6).

As presented in Fig. 7A and B, although all groups displayed a similar AAR% to the total LV, the percent of INF to AAR was substantially reduced by the three H_2_S donors at 72 h after reperfusion, among which, the DATS-MSN decreased the INF/AAR percent to the greatest extent. The echocardiography results (Fig. 7C) revealed that DATS-MSN most effectively preserved the cardiac function at 72 h after the infarction and reperfusion, presenting an increased ejection fraction and fractional shortening (Fig. 7D and E), as well as reduced LVIDd and LVIDs (Fig. 7F and G) when compared with the NaHS, DATS, and Vehicle groups. Moreover, the levels of H_2_S in plasma and myocardium remained elevated in the DATS-MSN group after the 72 h reperfusion process, but not in the NaHS and DATS groups (Fig. 7H and I).

### DATS-MSN Demonstrated Potential Myocardial Protective Advantages to GYY4137

**Figure 8:**
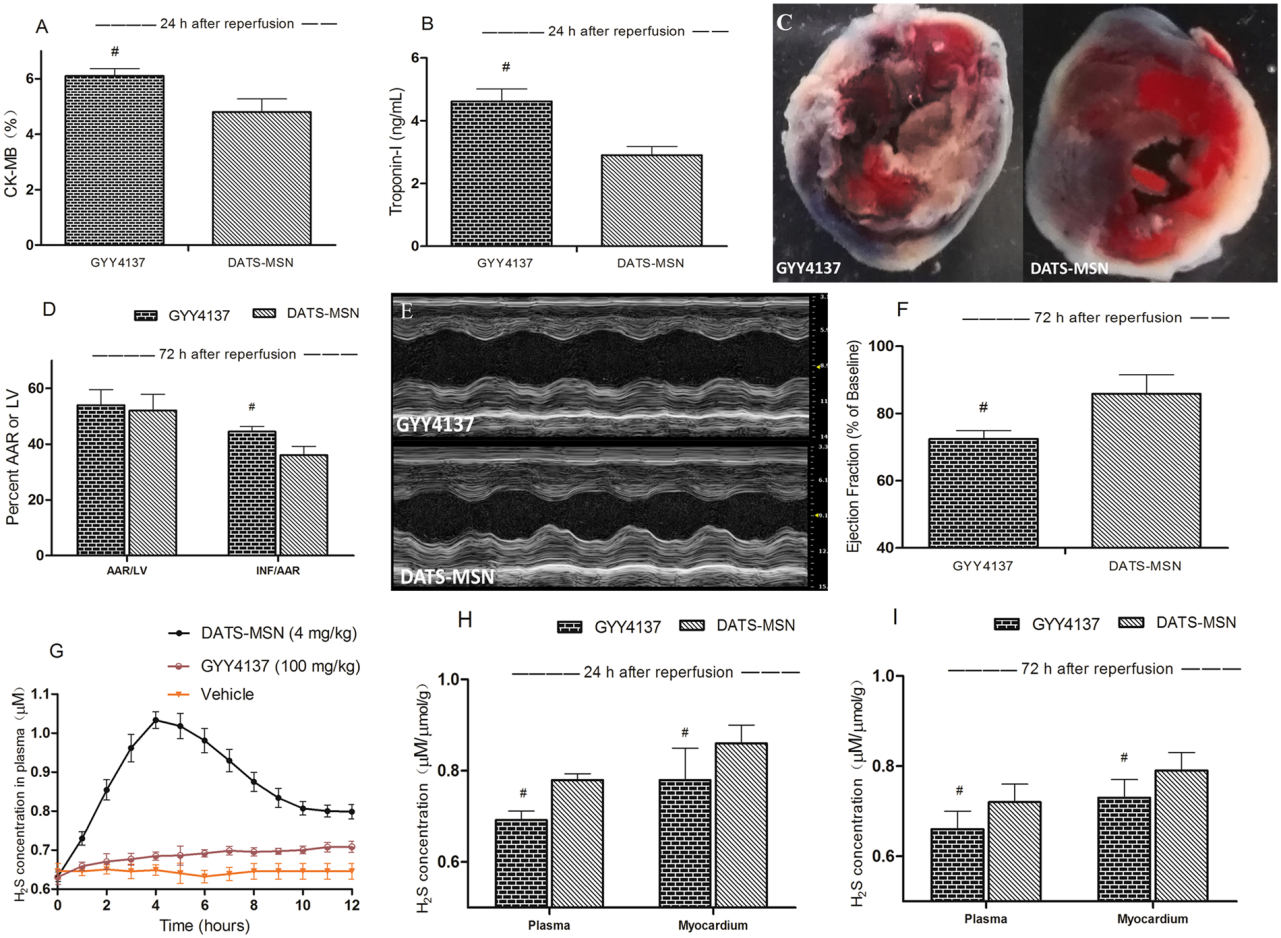
Myocardial injury and *in vivo* H_2_S levels evaluation at 24 h or 72 h after reperfusion and administration of GYY4137 and DATS-MSN. Changes in serum creatine kinase MB (CK-MB) (**A**) and Troponin-I (**B**) levels at 24 h after reperfusion; (**C**) Representative mid-myocardial cross sections of TTC-stained hearts and (**D**) percentage of area at risk (AAR) to total LV and infarction area (INF) to AAR; Representative M-mode images (**E**) and ejection fraction (**F**) from each group at 72 h after reperfusion; H_2_S levels during 12 h after reperfusion in plasma (**G**), and at 24 h (**H**) and 72 h (**I**) after reperfusion in plasma and in myocardium were measured. ^#^P < 0.05 compared with the DATS-MSN group (mean ± SEM, n = 6).

The CK-MB (Fig. 8A) and troponin I (Fig. 8B) levels were more effectively reduced by DATS-MSN compared with GYY4137 at 24 h after reperfusion, while INF/AAR was also more obviously reduced by the DATS-MSN at 72 h after reperfusion (Fig. 8C and D), with more preserved cardiac function (Fig. 8E and F). Meanwhile, DATS-MSN was also associated with superior anti-apoptosis ability than GYY4137 (Fig. S4). However, there was no obvious difference between groups in myocardial oxidative stress (Fig. S5) and inflammation (Fig. S6) assessment. In H_2_S releasing measurement, although GYY4137 presented a quite slow releasing pattern of H_2_S, H_2_S levels of which remained lower than DATS-MSN in 12 h after reperfusion in plasma (Fig. 8G); and at 24 h and 72 h after reperfusion in plasma and myocardium (Fig. 8H and I).

## Discussion

H_2_S has been shown to effectively reduce the infarct size, preserve cardiac function, and improve the survival rates in I/R injury models^1, 2^. To date, although several mechanisms of the cardioprotective effects of H_2_S have been elucidated including anti-apoptosis, anti-inflammatory, and antioxidant effects^4^, how the different H_2_S releasing patterns mediate its physiological effects remains unknown. Herein we present a novel slow-releasing H_2_S donor as an ideal platform for studying the slow release pattern of H_2_S both *in vitro* and *in vivo*. To clarify the advantage of the slow-releasing H_2_S donor with respect to myocardial protection, all of the drugs were administrated at the onset of reperfusion as post-conditioning. Ischemic post-conditioning has been previously shown to attenuate I/R injury; however, it has failed to easily translate into therapeutically useful cardioprotective strategies in clinical practice^14, 15^. In comparison, pharmacological post-conditioning may provide more clinical advantages, which would be fairly easy to be used in an intravenous formulation, and effectively cover the necessary time frame to mediate protection against lethal reperfusion injury^16^. H_2_S donors with the same possible H_2_S production administrated at the same time point (onset of reperfusion) can eliminate the majority of possible disturbances, and the effects of the different H_2_S releasing patterns are clearly presented.

H_2_S has been widely studied using *in vitro* models of cardiovascular diseases, demonstrating various significant cytoprotective effects against cell loss and cellular injury^1, 17^. In the present study, DATS-MSN manifested a superior cytoprotective effect compared with both NaHS and DATS. Along with the expansion of the reperfusion time, the protective advantage of DATS-MSN became increasingly more remarkable. Correspondingly, DATS-MSN also presented a more slowly releasing H_2_S pattern than NaHS and DATS, suggesting that the superior protective effects of DATS-MSN may be attributed to its slow-releasing pattern and long-term H_2_S effects *in vitro*.

In the *in vivo* I/R models, DATS-MSN also demonstrated superior cardioprotective effects over other H_2_S donors, associated with decreased CK-MB and troponin I levels and improved survival rates at 24 h after reperfusion, as well as elevated MAP levels and decreased arrhythmia scores in the I/R process. In contrast to the rapidly increased H_2_S concentrations in plasma associated with NaHS, DATS-MSN released H_2_S slowly and stably, and H_2_S remained elevated in plasma as well as in ischemic myocardium after 24 h of reperfusion. In addition, DATS increased the H_2_S concentration in plasma more rapidly than DATS-MSN, but slowly than NaHS, with plasma and myocardial H_2_S levels between the NaHS and DATS-MSN groups at 12 h following reperfusion. Therefore, with the same amount of S atoms, both the different releasing speeds of the H_2_S donors and the maintenance of the H_2_S levels are related to the extent of myocardial injury.

It was shown previously that H_2_S exerts anti-apoptotic effects in different organs, especially in I/R hearts^18^. In the present study, the DATS-MSN group exhibited a significantly decreased apoptosis index, suggesting that an anti-apoptotic mechanism may play an important role in the cardioprotective effects of the novel H_2_S donor. Multiple studies have reported that H_2_S exerts anti-apoptotic effects via the inactivation of caspases caused by I/R^19^. In this study, DATS-MSN administration was associated with H_2_S-mediated modulation through increasing the levels of Bcl-2, decreasing the levels of Bax, and reducing the activity of caspases-3. Although NaHS and DATS were associated with the same anti-apoptotic mechanisms, they were not as adequate as DATS-MSN due to their relatively short duration of efficacy.

It was found that oxidative stress plays an important role in the I/R injury, and H_2_S potently protects cells from oxidative stress through multiple mechanisms^20^. In the present study, the elevated levels of MDA were reduced to the greatest extent by DATS-MSN among the three H_2_S donors. In addition, DATS-MSN was also associated with the greatest preservation of antioxidant levels and activity (e.g., SOD, CAT, and GSH), greatly reinforcing the antioxidant defenses. DATS-MSN also effectively attenuated the level of myocardial inflammation following the I/R process. Ischemic myocardial neutrophils assessed by an MPO analysis and the level of inflammatory cytokines were all markedly reduced by the DATS-MSN administration compared to those observed in other H_2_S donor groups and the Vehicle control. The advantage of DATS-MSN over NaHS and DATS regarding the antioxidant and anti-inflammatory effects may also attribute to its slow release and long-term effects of H_2_S.

To further identify the advantage of the slow and stable release feature of DATS-MSN, an I/R experiment with a longer reperfusion time was performed. Due to the slow release ability of DATS-MSN, the H_2_S concentration in either plasma or myocardium remained elevated at 72 h after reperfusion, of which returned to the baseline levels in both the DATS and NaHS groups. Correspondingly, DATS-MSN also demonstrated superior cardioprotective effects over other H_2_S donors, with most reduced infarct size in the long-term reperfusion experiment. Furthermore, DATS-MSN was also superior regarding the preservation of cardiac function and preventing LV dilatation following an infarction; this could potentially reverse LV remodeling and ameliorate the development of heart failure after an infarction, similar to some other endogenous H_2_S donors^21^. The greater protective effects combined with expanded reperfusion time highlight the advantage of this slow-releasing H_2_S donor.

**Figure 9:**
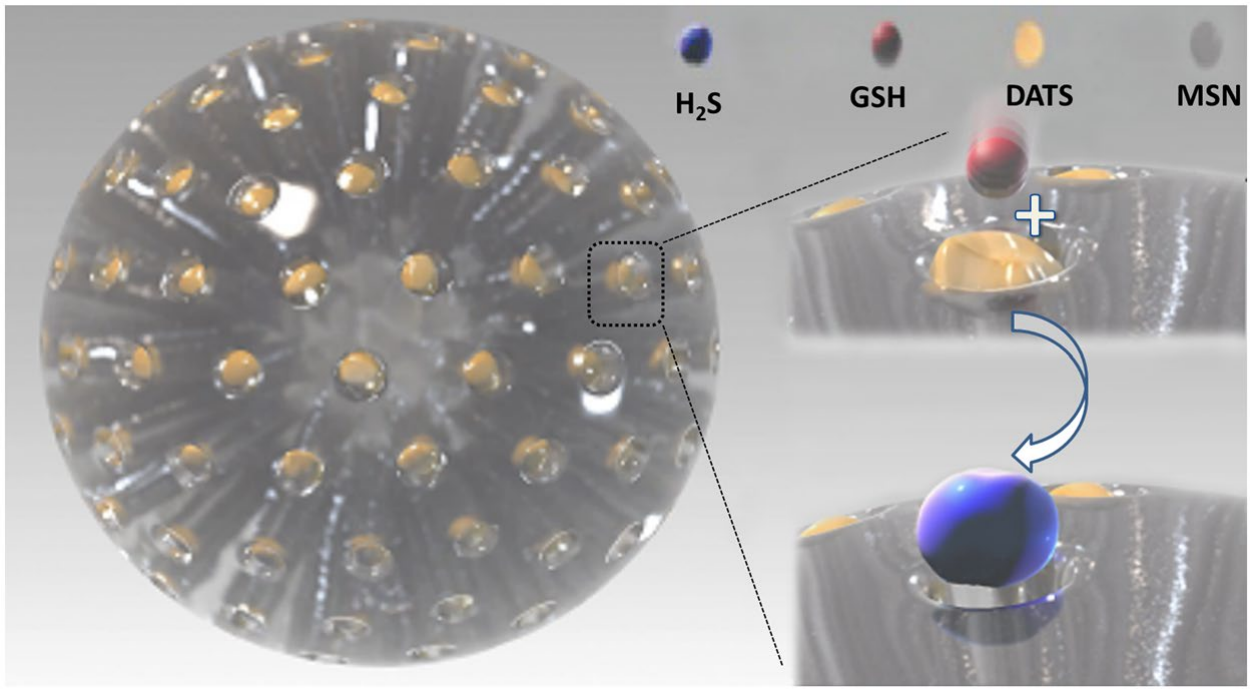
Schematic illustration of the mechanism of H_2_S slow-release from DATS-MSN. GSH molecules move into the mesopores to react with loaded DATS for H_2_S generation, after which H_2_S slowly releases into solution.

To date, the release pattern of H_2_S from relevant H_2_S donors has rarely been identified. NaHS is the most frequently applied donor in H_2_S studies; however, the extremely rapid release and loss pattern of this donor serves to limit its biofunctionality. H_2_S is easily transferred across respiratory membranes^22, 23^, thus, the residence time in tissues is relatively short for fast-releasing H_2_S donors, which limits their therapeutic potential and clinical application. In contrast, slow-releasing H_2_S donors may offer an accumulation of H_2_S over a long period, and they are more potent for providing therapeutic functions^3^. As previously reported, the slow-release mechanism of DATS-MSN could be due to GSH molecules moving into the mesopores and reacting with loaded DATS to generate H_2_S, after which the H_2_S is slowly released into solution and participates in the protective process (Fig. 9, Video 1). Moreover, the cardiovascular protective effects of DATS have also been found to be derived from H_2_S^9^. Therefore, the differences in the cardioprotective abilities between different H_2_S donors may be attributed to the different release patterns of H_2_S. In this study, DATS-MSN was associated with an overall superiority regarding its anti-apoptotic, antioxidant, and anti-inflammatory abilities over NaHS and DATS, contributing to the long-range effects of H_2_S, which can precisely mimic the physiological production and function of H_2_S.

Besides releasing pattern, the physiological effects of H_2_S also rely on its local effecting concentration. Sharing the same *in vivo* I/R model, time of administration and evaluating method, DATS-MSN presented some superior myocardial protective abilities than GYY4137, which may be attributed to its relatively high concentrations of H_2_S in plasma and myocardium. A satisfactory level of H_2_S over a long period conforms to an ideal H_2_S donor’s demand; DATS-MSN presented a more elevated H_2_S level than GYY4137 in the 72 h reperfusion process, which is a vital stage to myocardial preservation and LV remodeling. This may explain the potential advantages of DATS-MSN in myocardial injury protection. However, GYY4137 demonstrated comparable antioxidant and anti-inflammation effects to DATS-MSN, suggesting that it may involve additional protective mechanisms in GYY4137, which develops multiple cardioprotective effects by diverse mechanisms in a long time manner^24, 25^. Therefore, it is currently too early to conclude DATS-MSN exerting overall superiority to traditional slow-releasing H_2_S donors; actually it mainly provides us a novel slow-releasing alternative with new insight into control-releasing H_2_S based on nano-system, which has the potential to further regulate H_2_S releasing speed by adjusting structures of the nanoparticle^10, 26^.

In summary, we demonstrated that DATS-MSN as a novel slow-releasing H_2_S donor, can exert potent protective effects in rats and isolated cardiomyocytes following an I/R injury. Moreover, DATS-MSN exhibited superior cardioprotective effects in comparison to NaHS, DATS and GYY4137, which may be related to its specific show-releasing properties both *in vitro* and *in vivo*. Finally, DATS-MSN may primarily meditate the protective effects of H_2_S via various anti-apoptotic, antioxidant, and anti-inflammatory mechanisms. The present work provides a novel insight into how the H_2_S release patterns derived from different H_2_S donors affect their physiological functionality.

## Electronic supplementary material

Supporting Information

Releasing Mechanism of DATS-MSN
