# Supplementary material for: A Long-Term and Slow-Releasing Hydrogen Sulfide Donor Protects against Myocardial Ischemia/Reperfusion Injury
Source: Sci Rep. 2017 Jun 14;7:3541. doi: 10.1038/s41598-017-03941-0 (PMC5471203; doi:10.1038/s41598-017-03941-0)
Supplement: Supplementary file 1 — Supporting Information [file 41598_2017_3941_MOESM1_ESM.pdf]

Supporting Information

A Long-Term and Slow-Releasing Hydrogen Sulfide Donor Protects against Myocardial Ischemia/Reperfusion Injury

Xiaotian Sun[1†*], Wenshuo Wang[2†], Jing Dai[3], Sheng Jin[4], Jiechun Huang[1], Changfa Guo[2], Chunsheng Wang[2], Liewen Pang[1*], and Yiqing Wang[1*]

1 Department of Cardiothoracic Surgery, Huashan Hospital of Fudan University, Shanghai 200040, China

2 Department of Cardiac Surgery, Zhongshan Hospital of Fudan University and Shanghai Institute of Cardiovascular Diseases, Shanghai 200032, China

3 Department of Cadres Health Care, Third Hospital of Shijiazhuang, Shijiazhuang 050011, China

4 Department of Physiology, Hebei Medical University, Shijiazhuang 050017, China

† These authors contributed equally to this work.

***Corresponding author:**

Xiaotian Sun

Address: 12th, Wulumuqi Road, Shanghai, 200040, P. R. China

Email: sunxiaotian@huashan.org.cn; Telephone number: +862152887066

&

Liewen Pang

Address: 12th, Wulumuqi Road, Shanghai, 200040, P. R. China

Email: pangliewen@huashan.org.cn; Telephone number: +862152887066

&

Yiqing Wang

Address: 12th, Wulumuqi Road, Shanghai, 200040, P. R. China

Email: wangyiqing@huashan.org.cn; Telephone number: +862152887066

Preparation and Characterization of DATS-MSN

The synthesis of DATS-MSN was carried out by sol-gel method [1, 2]. Briefly, 10 mg mesoporous silica nanoparticles (MSN) and 8 mg diallyl trisulfide (DATS) were dispersed in 2 mL distilled water to form the mixture with a ratio of drug/carrier 0.8/1, which was stirred at room temperature for 12 h, followed by centrifugation (14000 rpm, 10 min). The nanoparticles obtained were washed with distilled water to remove DATS on the surface of MSN.

The exact diameters of synthesized nanoparticles were acquired by averaging size of 50 nanoparticles in transmission electron microscopy (TEM) images, which were also applied to confirm the frameworks and structures of MSN. Unloaded DATS was subtracted from initial total DATS to calculate the mass of DATS loaded in MSN. The drug loading content was calculated as mass of drug loaded in nanoparticles /mass of drug loaded nanoparticles*100 %.

TEM images (**Fig. S1**) indicated that the MSN particles are monodisperse with uniform size and regular mesopores. The diameter of MSN is ~ 225 ± 35 nm. The calculated drug loading contents of the samples are ~ 44.1 ± 0.1% (n = 3).

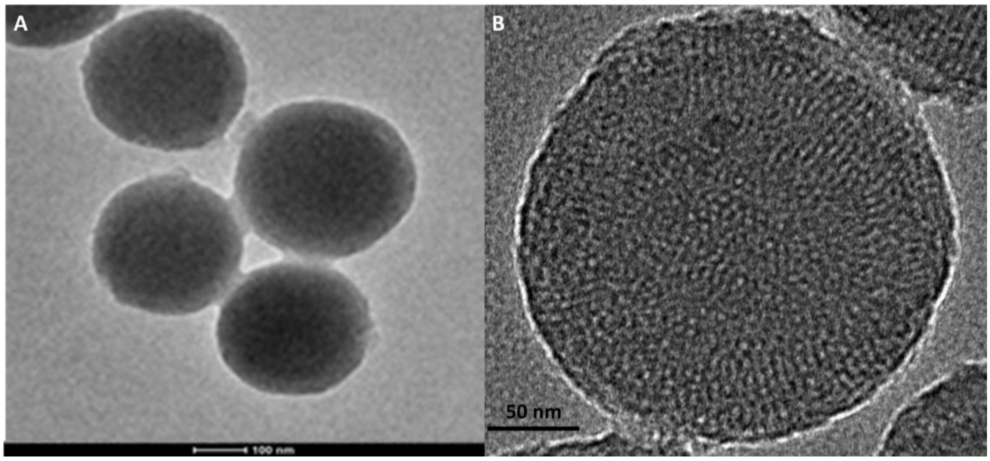

Figure S1. Transmission electron microscopy (TEM) images of particle diameter (A) and porous structure (B) of DATS-MSN.

Isolation and Culture of Primary Neonatal Cardiomyocytes

Primary neonatal cardiomyocytes were obtained from the cardiac tissues of newborn Sprague-Dawley rats (6 g, 24 h). Isolated cardiomyocytes were seeded at a density of $1*10^5$ cells/mL in Dulbecco's Modified Eagle's Medium/F12 (DMEM/F12, Gibco Invitrogen, CA, USA) supplemented with 10 % fetal bovine serum, 100 U/mL penicillin, 100 mg/mL streptomycin, and 100 mM 5-bromodeoxyuridine. Cells were cultured in a humidified incubator at 37 ℃ with 95% air and 5% CO_2 for 72 h.

Most Protective Concentrations of NaHS and DATS in Hypoxia/reoxygenation Procedure

The hypoxia/reoxygenation procedure was described in the main text. According to the different concentrations of NaHS or DATS added into the culture medium, each drug was divided into 11 subgroups: NaHS groups (0 to 100 μM, each 10 μM as gradient) and DATS groups (0 to 10 μg/mL, each 1 μg/mL as gradient). After the 4 h hypoxia and 6 h reoxygenation process, the cell viabilities in different groups were measured by CCK-8 method. Most protective concentrations of NaHS and DATS were selected. The results were expressed as the percentages relative to 0 control (n = 3).

As shown in **Fig. S2**, the most protective concentration of NaHS emerged to be at 80 μM, and DATS appeared to most effectively protect cardiomyocytes at the concentration of 5 μg/mL.

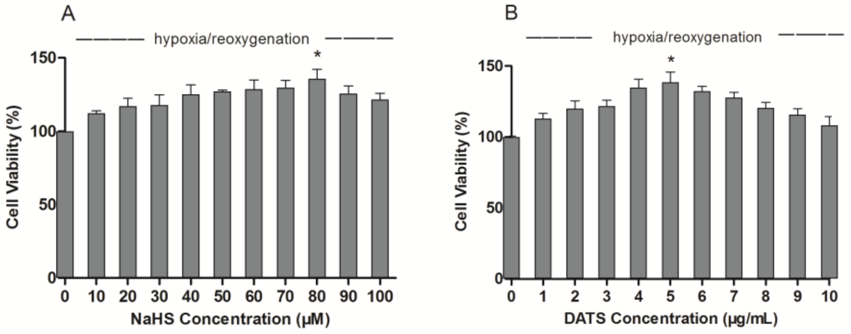

Figure S2. Comparisons of different concentrations of NaHS (A) or DATS (B) in protective effects from hypoxia/reoxygenation induced damage in rat cardiomyocytes. *: $P < 0.05$ compared with the 0 μM (A) or 0 μg/mL (B).

Most Protective Dosages of NaHS, DATS and GYY4137 in Ischemia and Reperfusion Protocol

According to the different dosages of NaHS, DATS or GYY4137 injected into the tail vein of rats, each drug was divided into 11 subgroups: NaHS groups (0 to 100 μmol/kg, each 10 μmol/kg as gradient), DATS groups (0 to 10 mg/kg, each 1 mg/kg as gradient) and GYY4137 groups (0 to 250 mg/kg, each 25 mg/kg as gradient). After 30 min ischemia and 24 h reperfusion, the rat blood samples were obtained, and creatine kinase MB (CK-MB) was measured to indicate myocardial injury by Anilytics (Gaithersburg, MD, USA). The most protective dosages of each H_2S donor were selected (n = 3).

As shown in **Fig. S3**, the most myocardial protective dose emerged to be of 30 μmol/kg for NaHS; 2 mg/kg for DATS and 100 mg/kg for GYY4137.

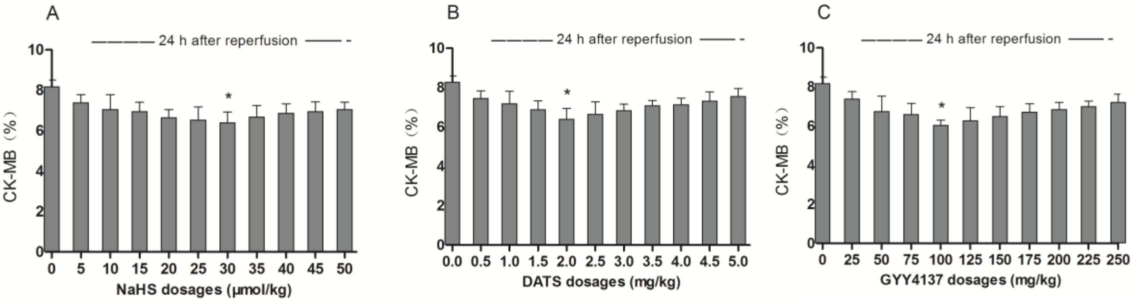

Figure S3. Comparisons of different dosages of NaHS (A), DATS (B) or GYY4137 (C) in protective effects from ischemia and reperfusion induced myocardial injury: creatine kinase MB (CK-MB) measurement. *: P < 0.05 compared with the 0 dose (same volume of PBS, Vehicle).

Measurement of H_2S Concentration in Cell Culture Medium, Plasma and Heart Tissues

H_2S concentrations in cell culture medium and in rat plasma were both measured to describe the release patterns of different H_2S donors. NaHS (80 μM) and DATS-MSN (50 μg/mL) were separately added into the culture medium, then the culture medium was collected at every hour from 1 to 6 h after administration. Rat plasma was collected from rat blood in Vehicle, NaHS, DATS and DATS-MSN, as well as GYY4137 groups at every hour from 0 h to 12 h after

reperfusion. Rat plasma and heart tissue in ischemia area were also collected at 24 h and 72 h after reperfusion before death. H_2S concentration was measured by high performance liquid chromatography (HPLC) method as described in the previous study [3]. H_2S concentration was determined using a standard curve of Na_2S (0-100 μM). The H_2S content in the heart tissues were quantified by protein content and expressed in proportion relative to Vehicle group. The protein content was measured by the BCA method mentioned above.

Arrhythmia Analysis and Scores

Ventricular tachycardia (VT) was defined as a run of four or more consecutive ventricular premature contractions. Ventricular fibrillation (VF) was defined as a ventricular rhythm in which one is unable to distinguish the individual QRS complexes of the ECG or to measure the rate. According to the cumulative duration of VT and VF, an arrhythmia score was graded as follows: 0: without arrhythmias; 1: less than three premature ventricular contractions per min; 2: greater than or equal to three premature ventricular contractions per min; 3: less than three episodes of VT per min; 4: greater than or equal to three episodes of VT per min or transient VF; and 5: frequent or sustained VF or death of the rat. The number corresponded to the most severe type of arrhythmia observed in each heart during the periods of ischemia and early reperfusion, and the scores were used for intergroup analysis of their severity.

Echocardiography

At 72 h after reperfusion, rats were anesthetized again by previous protocol and placed on a heating pad before sacrifice. Transthoracic echocardiography was performed using the Philips IE 33 system and a 12-4 MHz linear transducer (S12-4, Philips, AMS, NED). Left ventricular internal dimension in systole (LVIDs) and left ventricular internal dimension in diastole (LVIDd) were all obtained from the M-mode tracings. Ejection fraction (EF) and fractional shortening (FS) were also derived to evaluate

cardiac function. All echocardiographic measurements were performed by skilled observer blindly.

DATS-MSN Exerts Superior Anti-apoptosis Ability than GYY4137

As shown in **Fig. S4,** DATS-MSN was associated with superior anti-apoptosis ability than GYY4137 at 24 h after reperfusion.

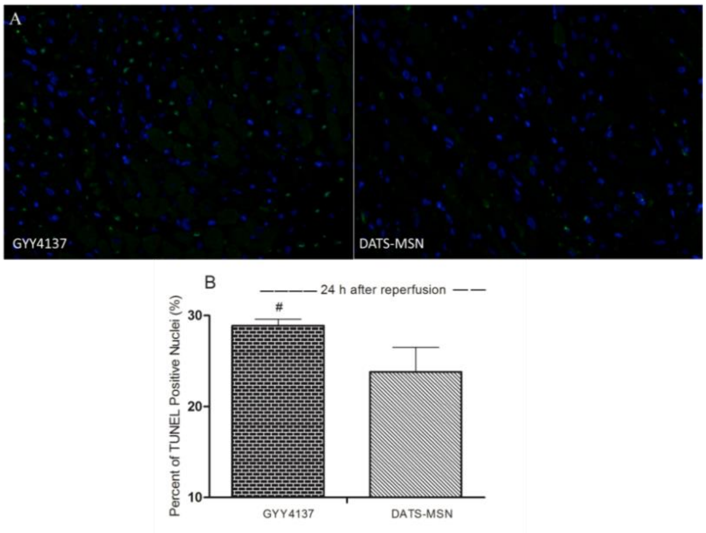

Figure S4. The changes of cardiomyocytes apoptosis after 24 h reperfusion and administration of GYY4137 and DATS-MSN: (A) TUNEL staining detected cardiomyocytes apoptosis and (B) the percentage of TUNEL positive cells to total cardiomyocytes. #: $P < 0.05$ compared with the DATS-MSN group (mean ± SEM, n = 6).

DATS-MSN Exerts Comparable Antioxidant and Anti-inflammation Ability to GYY4137

At 24 h after reperfusion, there was no obvious difference between GYY4137 and DATS-MSN groups in myocardial oxidative stress (**Fig. S5**) and inflammation (**Fig. S6**) evaluation.

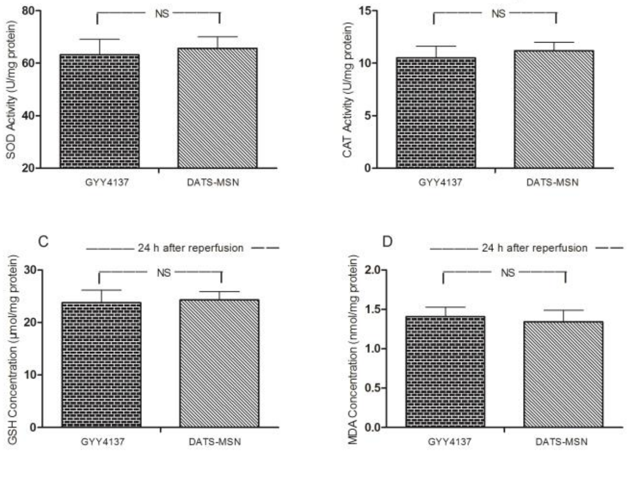

Figure S5. Levels of antioxidant defensive enzymes and malonydialdehyde (MDA) levels in heart tissues after ischemia/reperfusion injury in GYY4137 and DATS-MSN groups. (A) Superoxide dismutase (SOD) activities, (B) catalase (CAT) activities, (C) levels of glutathione (GSH) and (D) levels of MDA contents in ischemia heart tissue were evaluated after 30 min ischemia and 24 h reperfusion. No significant difference was observed between groups (mean ± SEM, n = 6).

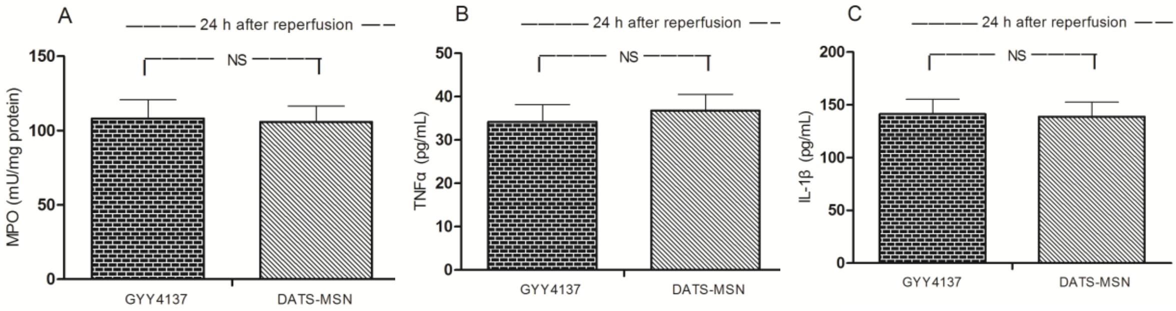

Figure S6. Myocardial inflammation levels after ischemia/reperfusion injury in GYY4137 and DATS-MSN groups: Myeloperoxidase (MPO) activity (A), TNF-α (B) and IL-1β (C) levels after 30 min ischemia and 24 h reperfusion. No significant difference was observed between groups (mean ± SEM, n = 6).

Video.1: Schematic illustration of the mechanism of H_2S slow-release from DATS-MSN. GSH molecules move into the mesopores to react with loaded DATS for H_2S generation, after which H_2S slowly releases into solution.

References

1. Slowing II, Trewyn BG and Lin VS. Mesoporous silica nanoparticles for intracellular delivery of membrane-impermeable proteins. *J Am Chem Soc* **129**, 8845-9 (2007).

2. H Tang, J Guo, Y Sun, et al. Facile synthesis of pH sensitive polymer-coated mesoporous silica nanoparticles and their application in drug delivery. *Int J Pharm* **421**, 388-96 (2011).

3. Sun, X, Kong, B and Wang W, et al. Mesoporous silica nanoparticles for glutathione-triggered long-range and stable release of hydrogen sulfide. *J Mater Chem B* **3**, 4451-7 (2015).
